# Bisphenol A Imprinted Electrochemical Sensor Based on Graphene Quantum Dots with Boron Functionalized g-C_3_N_4_ in Food Samples

**DOI:** 10.3390/bios13070725

**Published:** 2023-07-12

**Authors:** Haci Ahmet Deveci, Müge Mavioğlu Kaya, İnan Kaya, Bahar Bankoğlu Yola, Necip Atar, Mehmet Lütfi Yola

**Affiliations:** 1 Department of Nutrition and Dietetics, Faculty of Health Sciences, Gaziantep University, Gaziantep 27000, Turkey; h_ahmet_deveci@gantep.edu.tr; 2 Department of Molecular Biology and Genetic, Faculty of Arts and Sciences, Kafkas University, Kars 36000, Turkey; m.mavioglu@kafkas.edu.tr; 3 Department of Biology, Faculty of Arts and Sciences, Kafkas University, Kars 36000, Turkey; inankaya@kafkas.edu.tr; 4 Department of Engineering Basic Sciences, Faculty of Engineering and Natural Sciences, Gaziantep Islam Science and Technology University, Gaziantep 27000, Turkey; 5 Department of Chemical Engineering, Faculty of Engineering, Pamukkale University, Denizli 20000, Turkey; natar@pau.edu.tr; 6 Department of Nutrition and Dietetics, Faculty of Health Sciences, Hasan Kalyoncu University, Gaziantep 27000, Turkey

**Keywords:** bisphenol A, molecularly imprinting, nanocomposite, food analysis

## Abstract

A molecular imprinted electrochemical sensor based on boron-functionalized graphitic carbon nitride (B-g-C_3_N_4_) and graphene quantum dots (GQDs) was presented for selective determination of bisphenol A (BPA). In particular, by combining the selectivity and high stability properties, which are the most important advantages of molecular imprinted polymers, and the highly sensitive properties of GQDs/B-g-C_3_N_4_ nanocomposite, a highly selective and sensitive analytical method was developed for BPA analysis. Firstly, GQDs/B-g-C_3_N_4_ nanocomposite was characterized by using microscopic, spectroscopic, and electrochemical techniques. This novel molecular imprinted electrochemical sensor for BPA detection demonstrated a linearity of 1.0 × 10^−11^–1.0 × 10^−9^ M and a low detection limit (LOD, 3.0 × 10^−12^ M). BPA-imprinted polymer on GQDs/B-g-C_3_N_4_ nanocomposite also showed good stability, repeatability and selectivity in food samples.

## 1. Introduction

BPA is frequently encountered in the stocking and distribution of solid and liquid nutrients. In terms of its endocrinological effects, BPA has potential adverse effects on the receptors of hormones such as estrogen, androgen, and thyroid hormone. It has been shown that BPA has organ toxicity in experimental animals, and it has been reported that the tolerable daily dose of this molecule can be up to a maximum of 4 μg/kg bw. It is absorbed in a short time from the gastrointestinal tract and conjugated with UDP-glucuronic acid in the intestine and liver to form the BPA-glucuronide (BPA-gluc) metabolite, which is excreted through urine and feces [1]. In addition, BPA sulfate and unconjugated BPA metabolites in the urine were detected during saturation in the glucuronidation pathway of BPA [2] Various analyses have been carried out to minimize or eliminate the negative effects of BPA on health, along with restrictions on the use of BPA due to its risks to health [3]. Studies have reported that BPA is commonly found in human serum, urine, and breast milk, and it has been revealed that this exposure is at a global level. It is also widely used in the manufacture of cans for food preservation and the lining of jar lids. Nine percent of the BPA produced each year is used for the production of the lining material in cans. Analyses of real samples such as blood, urine, and hair have been performed in previous studies to determine the severity of BPA exposure from different sources in organisms [4]. For instance, chromatography [5], enzyme immobilization [6], the fluorescence method [7], and the colorimetric technique [6] was developed for BPA detection in real samples. However, these methods had some difficulties such as complex sample preparation steps and high-priced instrumentation. For these reasons, simple, sensitive, and rapid analytical methods such as nanosensors based on voltammetry are urgently needed for BPA detection. Especially, the development of electrochemical sensors in the field of current analysis can provide innovative contributions to avoid these effects while trying to determine the possible negative effects of BPA on both humans and other living things.

g-C_3_N_4_ is a polymeric two-dimensional nanomaterial with superior surface features and quantum properties providing increased sensitivity and performance of the developed electrochemical biosensors/sensor [8]. In addition, it has high thermal and chemical stability and biocompatibility, indicating g-C_3_N_4_ as an suitable nanomaterial for designing stable and selective biosensor interfaces [9]. However, its insufficient areas and fast electron recombination restrain its significant applications [10]. Several modification techniques such as g-C_3_N_4_ doping with sulfur and phosphorus were presented to enhance the effective surface area and g-C_3_N_4_’s electron migration [11]. In addition, g-C_3_N_4_ doping with graphene quantum dots can provide more active areas and the facilitation of the separation of photogenerated e^−^-h^+^ pairs [12]. This situation explains that doping treatment with sulfur and phosphorus elements and graphene quantum dots can be effective techniques for modification of g-C_3_N_4_ in terms of BPA detection ability.

Boron as a dopant element can provide many unsaturated areas and efficient C–N interactions, causing the enhancement of electron transfer and charge excitation [13]. In this context, it has been proven that the complex formed by the coordinate covalent bond between boron acting as a Lewis acid and g-C_3_N_4_ acting as a Lewis base provides an important catalytic activity in electrochemical sensor applications [14]. In addition, these strong interactions between the components in the complex create stable sensor signals, and as a result, it is possible to prepare sensors with high repeatability and stability [15]. Graphene quantum dots as semiconductor nanocrystals 1–15 nm in diameter have been attracting attention in electrochemical sensor construction [16]. Graphene quantum dots are mainly composed of sp^2^ hybridized carbon and are mainly in crystalline form. They are highly luminous, biocompatible, dispersible in a range of solvents, and generally non-toxic. Graphene quantum dots are non-toxic and biologically inert materials that have attracted worldwide attention from academia and industry because they are chemically and physically stable due to their inherent inert carbon properties. They also have excellent absorption properties for increasing nanocomposites’ absorption bands [17]. Moreover, graphene quantum dots as electron acceptors can enhance electron transfer and decrease charge recombination [18].

In molecular imprinting technology, after the target molecule and functional monomer interact in the preliminary stage, the polymerization process is initiated by adding a crosslinker molecule to the medium. The resulting polymer has a structure that also includes a template molecule. By removing the template molecule from this polymer, molecularly imprinted polymers (MIPs) specific to the template molecule, which have binding sites in the form of template molecules, are obtained. MIPs are highly durable due to their high mechanical strength, resistance to heat and pressure, physical strength, and high stability in the presence of extreme conditions such as acids, bases, metal ions, and organic solvents [19,20]. In MIP-based sensor technology, polypyrrole is one of the most widely used conductive polymers. High sensitivity, fast response time, and repeatability are especially achieved in sensors using this type of conductive polymer [21,22].

MIP-based sensors have been developed and applied to BPA determination in the literature. For example, ferrocenyl-based MIP in the presence of supercritical CO_2_ was synthesized and applied to BPA detection, and a linearity of 4.7 × 10^−9^–8 × 10^−9^ M was obtained [23]. In another study, Chai and Kan developed an MIP-based electrochemical sensor including gold–polythionine nanowires for BPA recognition, and a LOD of 3.8 × 10^−8^ M was calculated, providing the development of a sensitive sensor [24]. In addition, a MIP-based BPA sensor was designed by electropolymerization in the presence of pyrrole and applied to canned oil samples after characterization of the sensor by various microscopic techniques. A LOD of 4.0 × 10^−8^ M was obtained [25]. According to these studies, molecular imprinting technology is used extensively in the analysis of harmful agents such as BPA in food samples.

This paper indicates a novel molecular imprinted sensor for BPA analysis based on graphene quantum dots with boron-functionalized g-C_3_N_4_ in orange juice sample. The produced nanocomposite provided fast electron transfer and excellent electrochemical catalytic performance towards BPA target molecules. Especially, the incorporation of boron and graphene quantum dots enhanced the electrochemical performance of the designed BPA-imprinted electrochemical sensor in comparison with free g-C_3_N_4_. In this context, it has been proven that more sensitive BPA analysis can be performed, thanks to the developed sensor in this study, compared to other BPA analyses. In addition, by developing an environmentally and human-friendly nanocomposite, BPA analysis in food samples can be made more reliably and practically. Moreover, due to the high mechanical strength features of MIPs, a stable sensor design, which can be used for a long time compared to other BPA sensors, has been realized. Hence, this seminal study contributes to the literature in terms of healthier food consumption by presenting a highly selective, repeatable, and sensitive BPA analysis technique.

## 2. Experimental

### 2.1. Chemicals and Apparatus

BPA (purity of 99.99%), hydroxyphenol (HDP, purity of 99.99%), dopamine (DOP, purity of 99.99%), 4-nitrophenol (4-NIT, purity of 99.99%), ethanol (ETH, purity of 99.99%), bisphenol S (BPS, purity of 99.99%), melamine (MEL, purity of 99.00%), boron trioxide (B_2_O_3_, purity of 99.00%), citric acid (CA, purity of 99.00%), thiourea (purity of 99.00%) and pyrrole (Py, purity of 99.00%) monomer were obtained from Sigma-Aldrich. Phosphate-buffered saline (pH 7.0, PBS) (0.1 M) was chosen as a supporting electrolyte.

TEM, XRD, AFM, and XPS techniques were applied for the determination of structural analyses of g-C_3_N_4_, B-g-C_3_N_4_, and GQDs/B-g-C_3_N_4_ nanocomposite (see Supplementary Data File for apparatus).

### 2.2. Production of g-C_3_N_4_, B-g-C_3_N_4_, and GQDs/B-g-C_3_N_4_ Nanocomposite

After the transportation of MEL (10.0 g) in a crucible incubator, the incubator was transferred to a furnace and calcined at 600 °C for 5 h (5.0 °C min^−1^). Then, pure g-C_3_N_4_ was obtained as a yellowish powder [26]. After that, g-C_3_N_4_ (250.0 mg) was powdered with B_2_O_3_ (1.25 g). After the calcination of the mixture at 400 °C for 4 h (5.0 °C min^−1^), B-g-C_3_N_4_ was cooled to 25 °C and washed with ultrapure water at 30 °C.

After the dissolving CA (0.70 g) and thiourea (0.80 g) in ultrapure water (25.0 mL), the mixture was stirred for 45 min. After the transfer of the clear solution into a stainless autoclave (100.0 mL), the heating treatment was performed at 200 °C for 3 h, providing graphene quantum dots (GQDs). The impregnation technique was applied to the preparation of GQDs/B-g-C_3_N_4_ nanocomposite. B-g-C_3_N_4_ (300.0 mg) was mixed with an aqueous solution of GQDs (0.20 mg mL^−1^, 10.0 mL). After the stirring treatment for 24 h, filtration was conducted to remove the liquid phase. The obtained product (GQDs/B-g-C_3_N_4_) was dried at 80 °C for 18 h and washed with ultrapure water [27].

### 2.3. Production of GQDs/B-g-C_3_N_4_ Modified Glassy Carbon Electrode (GQDs/B-g-C_3_N_4_/GCE)

The procedure of surface cleaning of GCE (a geometric area of 0.070 cm^2^) was performed according to our previous study [28]. The clean GCE was modified with GQDs/B-g-C_3_N_4_ nanocomposite solution (30.0 μL, 0.4 mg mL^−1^) by dropping on an electrode surface, and an infrared heat lamp was applied to GCE modified with GQDs/B-g-C_3_N_4_ to remove the solvent for 15 min, providing GQDs/B-g-C_3_N_4_/GCE. g-C_3_N_4_- and B-g-C_3_N_4_-modified GCE surfaces (g-C_3_N_4_/GCE and B-g-C_3_N_4_/GCE) were formed by the method described above.

### 2.4. Development of BPA-Imprinted Sensor and BPA Removal

**Scheme 1:**
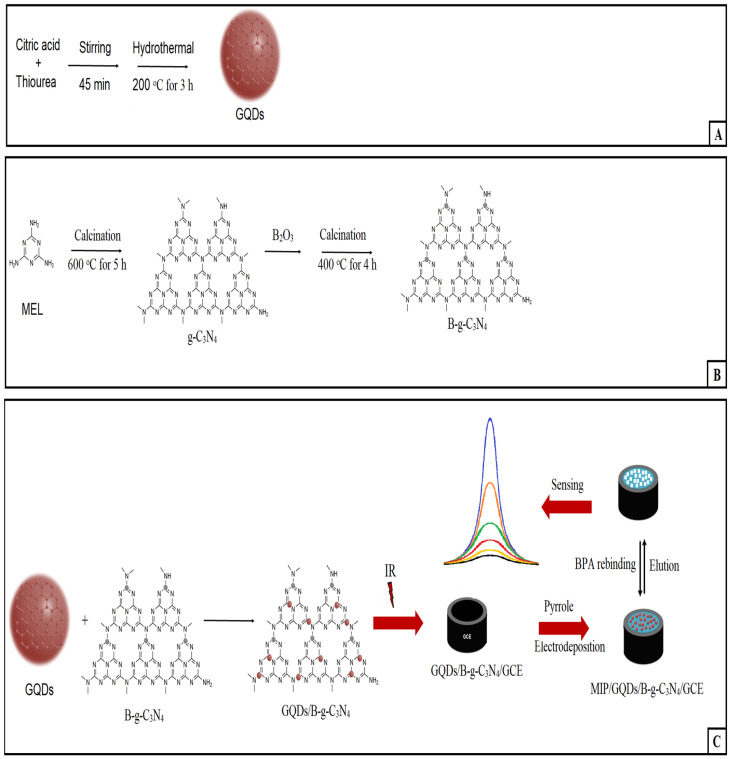
Preparation (**A**) GQDs, (**B**) B-g-C_3_N_4_ and (**C**) BPA-imprinted sensor.

Electrochemical molecular imprinting was the process of polymerization between the template (BPA) molecule and a monomer (Py) containing functional groups similar in shape and size to the template (BPA). Then, after the template molecule (BPA) was removed from the polymeric matrix with 1.0 M NaCl, a three-dimensional cavity, which was complementary to the template molecule (BPA) in terms of shape and size, was obtained. Thus, the developed polymeric matrix showed high selectivity towards BPA. For the experimental procedure, 100.0 mM Py in 0.1 M PBS (pH 7.0) including 25.0 mM BPA was transferred into the electrochemical cell to produce BPA-imprinted GQDs/B-g-C_3_N_4_/GCE (MIP/GQDs/B-g-C_3_N_4_/GCE). Then, 25 continuous cycles were performed on GQDs/B-g-C_3_N_4_/GCE in a range of +0.00 to +1.00 V. The method described above was applied for the production of BPA non-imprinted GQDs/B-g-C_3_N_4_/GCE (NIP/GQDs/B-g-C_3_N_4_/GCE) to demonstrate imprinting selectivity. Scheme 1 demonstrates the preparation procedure of the MIP sensor and the composite. For selectivity studies, selectivity coefficient (k) and relative selectivity coefficient (k′) values were calculated according to Equation (1).

k = ∆I_BPA_/∆I_interfering chemical_ and k′ = k_MIP_/k_NIP_ (1)

For desorption experiments, NaCl solution (10.0 mL, 1.0 M) was transferred into a shaker bath system. Then, the developed MIP electrodes were immersed into this bath system and shaken for 20 min.

### 2.5. Sample Preparation

The mixture of the obtained orange juice sample (10.0 mL) from the supermarket in Gaziantep, Turkey, and ethanol (10.0 mL) was put into a tube under strong mixing treatment for 1 min. After centrifugation for 10 min at 5000 rpm, the upper phase was collected and spiked with a fine syringe. After the dilution with 0.1 M, pH 7.0 PBS, the diluted sample was added into electrochemical cell.

## 3. Results and Discussion

### 3.1. Characterizations of g-C_3_N_4_, B-g-C_3_N_4_, and GQDs/B-g-C_3_N_4_ Nanocomposite

**Figure 1:**
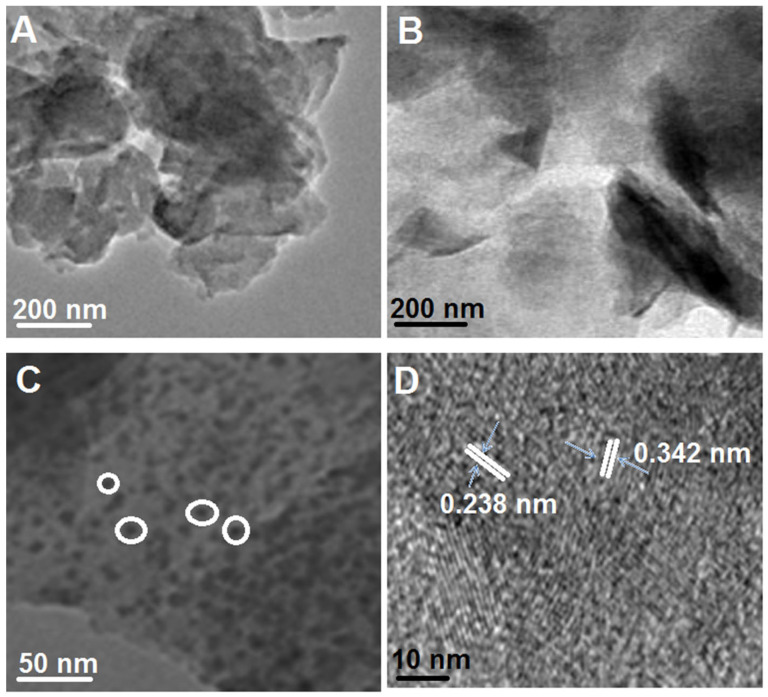
TEM images of (**A**) g-C_3_N_4_, (**B**) B-g-C_3_N_4_, (**C**) GQDs/B-g-C_3_N_4_, (**D**) high-resolution TEM image of GQDs/B-g-C_3_N_4_.

**Figure 2:**
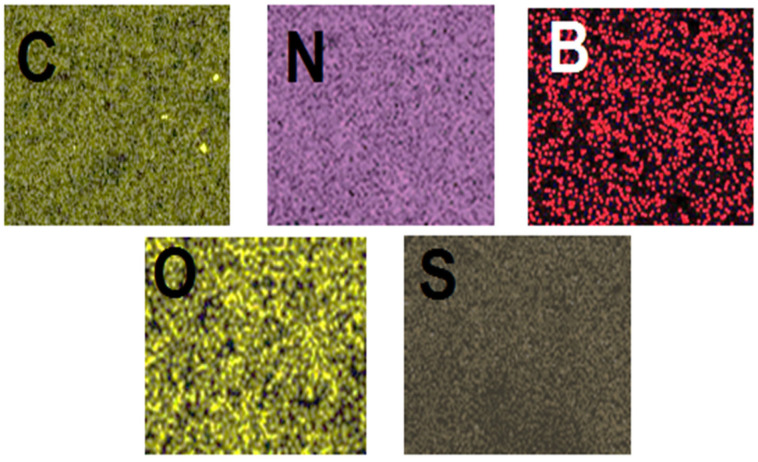
The element mapping of GQDs/B-g-C_3_N_4_ nanocomposite (C: carbon; N: nitrogen; B: boron; O: oxygen; S: sulfur).

The graphene quantum dots with boron-functionalized g-C_3_N_4_ (GQDs/B-g-C_3_N_4_) was prepared by an impregnation technique providing the enhancement of electrochemical activity. Firstly, TEM images were obtained for investigation of the morphological structure. According to Figure 1A, B, g-C_3_N_4_ with a layered structure and the boron doping treatment could not alter the morphological structure having g-C_3_N_4_. After the preparation of graphene quantum dots with boron-functionalized g-C_3_N_4_, small black dots in circles of 2–5 nm in diameter confirmed the presence of B-g-C_3_N_4_ on GQDs, indicating successful synthesis of the nanocomposite (Figure 1C). Finally, a high-resolution TEM image (Figure 1D) showing GQDs/B-g-C_3_N_4_ with a lattice space of 0.342 nm was attributed to the (002) crystal plane of the nanocomposite [27]. The lattice spacing distance of 0.238 nm was related to the lattice spacing distance of graphite, which provided carbon atoms of GQDs with sp^2^ bonded graphitic structures [29]. In addition, element mapping (Figure 2) of the GQDs/B-g-C_3_N_4_ nanocomposite verified the successful doping treatment, including graphene quantum dots with boron-functionalized g-C_3_N_4_.

**Figure 3:**
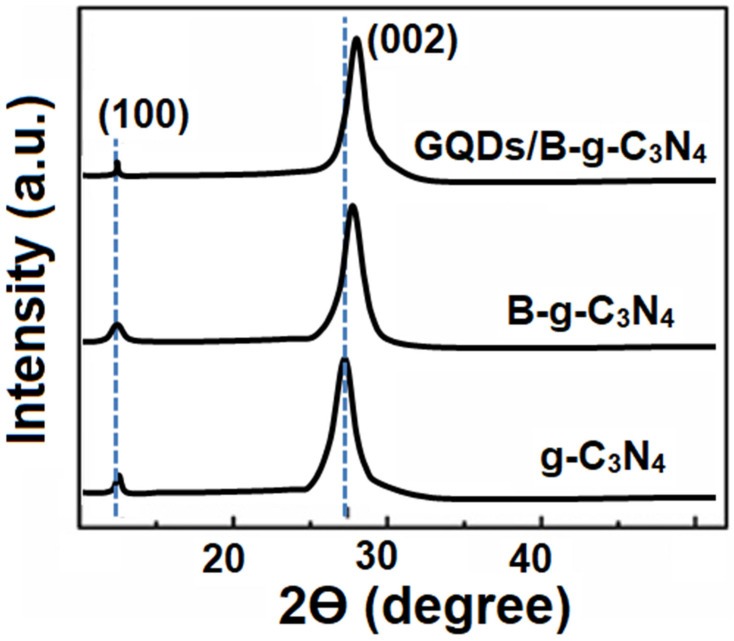
XRD patterns of g-C_3_N_4,_ B-g-C_3_N_4_, and GQDs/B-g-C_3_N_4_ nanocomposites.

The crystal structures of g-C_3_N_4_, B-g-C_3_N_4_, and GQDs/B-g-C_3_N_4_ were investigated by XRD (Figure 3). Two obvious XRD peaks at 12.89° and 27.64° corresponded to (100) and (002) planes, respectively [30]. A small XRD peak shift at 27.64° confirmed the boron doping, showing the modifications of lattice parameters and planar strains owing to the deficiency on the g-C_3_N_4_ structure [31]. In addition, due to the small concentration of GQDs and their dispersion, there was no XRD peak attributed to graphene quantum dots [18].

**Figure 4:**
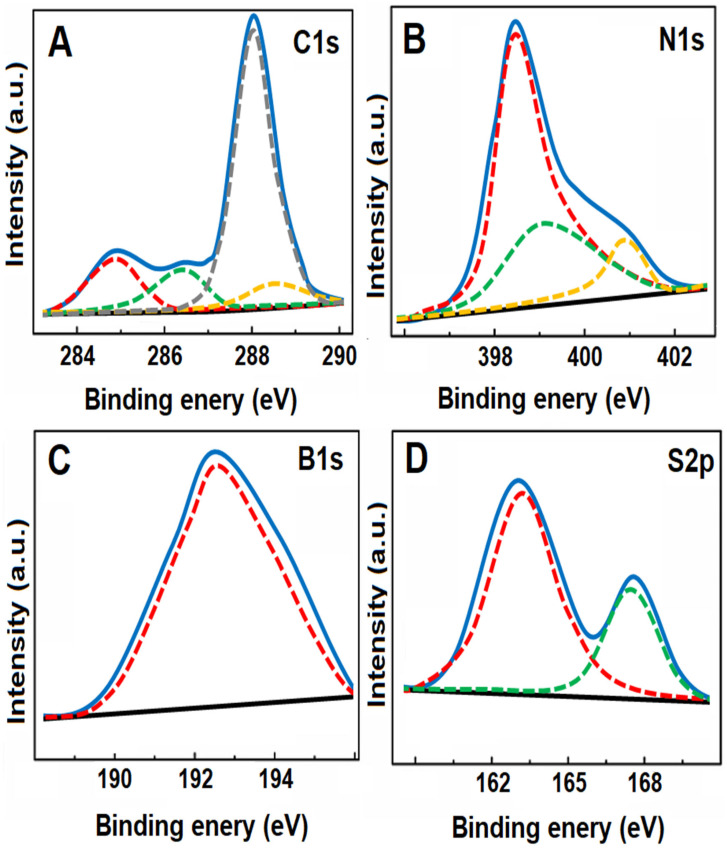
High-resolution XPS spectrums of (**A**) C1s, (**B**) N1s, (**C**) B1s, and (**D**) S2p of GQDs/B-g-C3N4 nanocomposite.

C1s, N1s, B1s, and S2p high-resolution XPS spectrums of GQDs/B-g-C_3_N_4_ nanocomposite are given in Figure 4. According to Figure 4A showing the C1s high resolution spectrum, four XPS peaks at 285.09, 286.79, 288.73, and 289.19 eV were obtained, attributing to –C=C–, –C–N_x_, and sp^2^-bonded –N=C–N– and –C=O–, respectively [32]. The novel XPS peak at 289.19 eV also resulted from the interaction between carbonyl and carboxyl groups, providing the successful introduction of GQDs into the B-g-C_3_N_4_ surface [33]. In addition, three XPS peaks at 398.73, 400.19, and 401.83 eV corresponded to –C–N=C– and sp^3^-bonded –N–(C)_3_ and –C–N–H– groups, respectively [34] (Figure 4B). The XPS peak at 193.18 eV was related to –B–N bonds on the XPS spectrum of B1s (Figure 4C) [35]. Finally, the S2p XPS spectrum demonstrated S2p3/2 and S2p1/2 peaks at 163.87 and 168.76 eV, corresponding to the presence of thiophene and –S=O–, respectively (Figure 4D) [33]. Especially, the presence of sulfur or sulfur oxide in the nanocomposite could cause higher charge density, providing electrochemical activity. Thus, XPS results verified the successful production of the GQDs/B-g-C_3_N_4_ nanocomposite.

**Figure 5:**
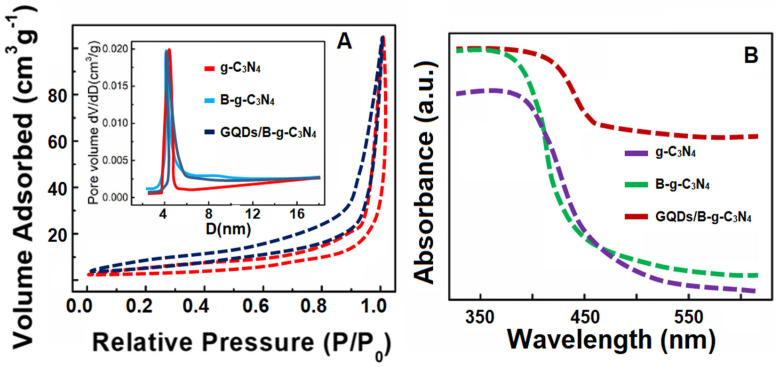
(**A**) N_2_ adsorption-desorption isotherms of g-C_3_N_4_, B-g-C_3_N_4_, and GQDs/B-g-C_3_N_4_ nanocomposite and (**B**) UV-Vis diffuse reflectance spectra of g-C_3_N_4_, B-g-C_3_N_4_, and GQDs/B-g-C_3_N_4_ nanocomposite.

Figure 5A shows N_2_ adsorption–desorption isotherms of g-C_3_N_4_, B-g-C_3_N_4_, and GQDs/B-g-C_3_N_4_ nanocomposites. The specific surface areas of g-C_3_N_4_, B-g-C_3_N_4_, and GQDs/B-g-C_3_N_4_ nanocomposite were calculated to be 15.96, 21.78, and 26.09 m^2^ g^−1^, respectively. The high surface area value could contribute to the alteration in pore-size distribution, providing more electrochemical active areas [36]. The UV–Vis DRS technique (Figure 5B) was employed for investigations of photon absorption properties of the nanomaterials. There was no absorption shift on the B-g-C_3_N_4_ spectrum in comparison with g-C_3_N_4_. Nonetheless, owing to the incorporation of GQDs into the nanocomposite, GQDs/B-g-C_3_N_4_ revealed an obvious red shift, facilitating more e^−^/h^+^ pairs [27]. Thus, it was concluded that the improved electrochemical activity was ensured via the synthesis of the GQDs/B-g-C_3_N_4_ nanocomposite.

### 3.2. Electrochemical Characterizations of g-C_3_N_4_, B-g-C_3_N_4_, and GQDs/B-g-C_3_N_4_ Nanocomposite-Modified Electrodes

**Figure 6:**
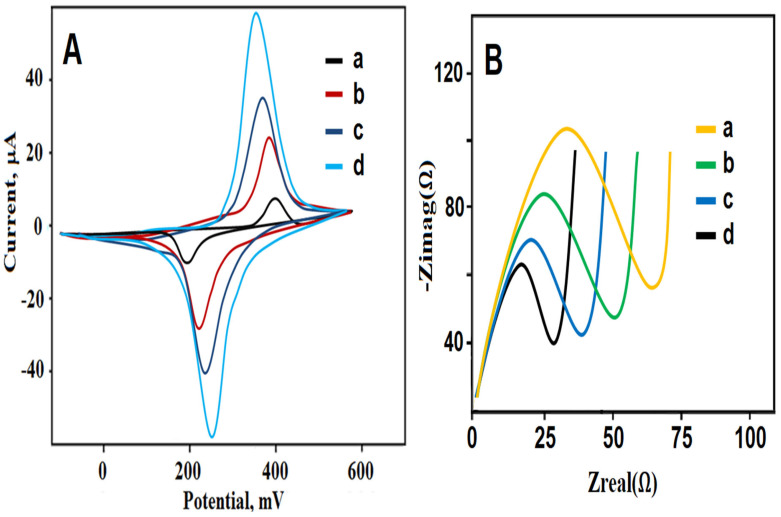
(**A**) CV curves and (**B**) EIS responses at (a) bare GCE, (b) g-C_3_N_4_/GCE, (c) B-g-C_3_N_4_/GCE, (d) GQDs/B-g-C_3_N_4_/GCE (redox probe: 5.0 mM [Fe(CN)_6_]^3−/4−^ containing 0.1 M KCl).

The electrochemical performance properties of g-C_3_N_4_, B-g-C_3_N_4_, and GQDs/B-g-C_3_N_4_ nanocomposite-modified electrodes were investigated via CV and EIS (Figure 6A). Electrochemical signals on g-C_3_N_4_/GCE (curve b of Figure 6A) appeared more prominent in comparison with bare GCE (curve a of Figure 6A) because of the specific surface area of carbon nitride material and its quantum effects. After boron loading on g-C_3_N_4_, since electron flow occurred more easily on the electrode surface, the improved electrochemical signals were observed on B-g-C_3_N_4_/GCE (curve c of Figure 6A) [11]. Lastly, the GQDs/B-g-C_3_N_4_ nanocomposite demonstrated the highest electrochemical performance owing to more adsorption sites and synergistic effects between GQDs and B-g-C_3_N_4_ (curve d of Figure 6A) [12].

According to EIS measurements (Figure 6B), the charge transfer resistance (*R_ct_*) values were determined to be 65 ohm for bare GCE (curve a), 45 ohm for g-C_3_N_4_/GCE (curve b), 35 ohm for B-g-C_3_N_4_/GCE (curve c), and 20 ohm for GQDs/B-g-C_3_N_4_/GCE (curve d). Thus, in accordance with the CV results, the most efficient electrochemical reaction occurred on GQDs/B-g-C_3_N_4_/GCE.

### 3.3. Fabrication of BPA-Imprinted Polymer on GQDs/B-g-C_3_N_4_/GCE

Twenty-five continuous cycles were scanned on GQDs/B-g-C_3_N_4_/GCE in the +0.00–+1.00 V range, and current responses at about +0.80 V were constantly followed. The continuous decrease in these signals with the increase in the number of scans proved that BPA-imprinted polymers were formed on GQDs/B-g-C_3_N_4_/GCE (Figure S1A in Supplementary Materials).

Measurements were performed in the presence of 0.5 nM BPA and without BPA to show the electrochemical performances of the prepared MIP and NIP electrodes and how important molecular imprinting technology is for BPA analysis. Curve a of Figure S1B shows no electrochemical signal in only PBS solution. According to curve c of Figure S1B and curve b of Figure S1B, the molecular imprinting technique created BPA molecule-specific nano-cavities on GQDs/B-g-C_3_N_4_/GCE for BPA analysis, and more measurable signals were obtained in the presence of 0.5 nM BPA.

Lastly, several BPA-imprinted electrodes such as MIP/bare GCE (curve a of Figure S1C), MIP/g-C_3_N_4_/GCE (curve b of Figure S1C), MIP/B-g-C_3_N_4_/GCE (curve c of Figure S1C), and MIP/GQDs/B-g-C_3_N_4_/GCE (curve d of Figure S1C) were prepared. After applying these prepared MIP electrodes to 0.5 nM BPA solution, MIP/GQDs/B-g-C_3_N_4_/GCE electrode was confirmed to be the most applicable electrode for BPA analysis from real samples. In addition, an equal transfer of protons and electrons in the electro-oxidation mechanism of BPA occurred on GQDs/B-g-C_3_N_4_/GCE (Figure S2) [37].

The surface thicknesses of bare GCE (Figure S3A) and BPA-imprinted polymer film on GQDs/B-g-C_3_N_4_/GCE (Figure S3B) were calculated as 5.03 ± 0.19 and 23.11 ± 0.45 nm, respectively, by AFM and the electropolymerization on GQDs/B-g-C_3_N_4_/GCE was performed successfully.

### 3.4. Optimization Studies

Optimization studies and the results are given in Supplementary Data (Figure S4).

### 3.5. Quantification Limit (LOQ) and LOD Values

**Figure 7:**
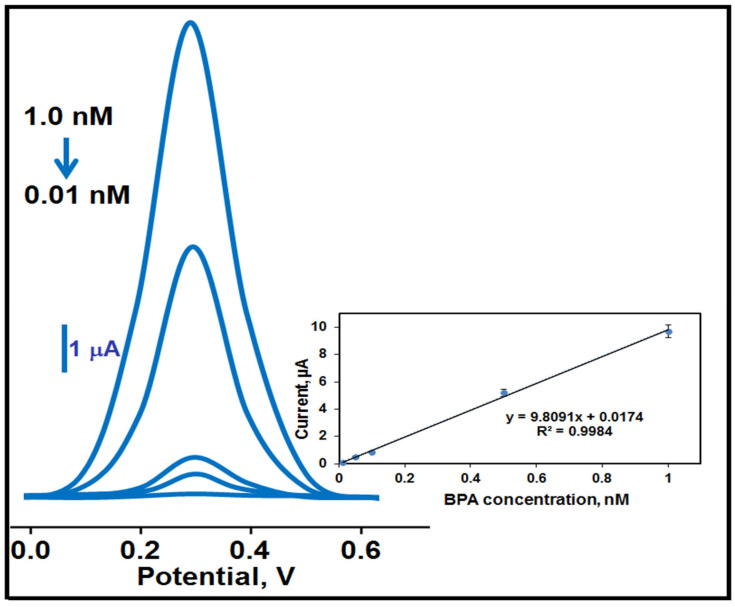
DPVs with different BPA amounts at MIP/GQDs/B-g-C_3_N_4_/GCE in pH 7.0 of PBS.

The calibration curve [y(µA) = 9.8091 × (C_BPA_,nM) + 0.0174, (R² = 0.9984)] was obtained on MIP/GQDs/B-g-C_3_N_4_/GCE by using BPA amounts and current signals (Figure 7), and LOQ and LOD values were computed as 1.0 × 10^−11^ M and 3.0 × 10^−12^ M, respectively (see Supplementary Data for the equations). In addition, Table 1 indicates the sensitivity features of MIP/GQDs/B-g-C_3_N_4_/GCE in comparison with the existing methods for BPA detection. Firstly, we successfully designed a sensor with satisfactory results compared to the literature. Since we produced GQDs/B-g-C_3_N_4_ nanocomposites by using impregnation and hydrothermal techniques in sensor preparation, we produced an environmentally friendly and harmless analytical method for BPA detection. In addition, a high efficiency GQDs/B-g-C_3_N_4_ nanocomposite with zero waste generation was produced. As a result, the BPA level in food can be followed more quickly and efficiently, leading to safer food consumption.

### 3.6. Recovery Assessment

For the recovery experiments, first of all, the orange juice sample, which was explained in detail in the sample preparation section, was transferred to four small experimental tubes in equal volumes. Standard BPA solutions at increasing concentrations were added to the other three tubes except for the first tube, and these 4 experimental tubes were finally diluted with 0.1 M, pH 7.0 PBS to equal volume. Then, these 4 tubes containing BPA in different concentrations were analyzed with the developed sensor. According to Table 2, the values close to 100% proved a high recovery. In addition, these results showed that the developed MIP-based sensor detects BPA in orange juice samples with high selectivity, and the interfering components do not negatively affect the high selectivity of the developed MIP-based sensor.

### 3.7. Selectivity, Repeatability, and Stability Performances of MIP/GQDs/B-g-C_3_N_4_/GCE

For selectivity experiments of MIP/GQDs/B-g-C_3_N_4_/GCE, 5 chemical agents with similar physical and chemical properties were detected (HDP, DOP, 4-NIT, ETH, and BPS). The electrochemical signals (µA) were obtained for BPA, HDP, DOP, 4-NIT, ETH, and BPS on MIP/GQDs/B-g-C_3_N_4_/GCE and NIP/GQDs/B-g-C_3_N_4_/GCE (Figure S5A,B). These electrochemical signals are given on Table S1, including k and k′ values. It was concluded that MIP/GQDs/B-g-C_3_N_4_/GCE was 10.00, 13.33, 20.00, 40.00, and 100.00 times more selective for BPA than HDP, DOP, 4-NIT, ETH, and BPS, respectively, because of specific nano-cavities of BPA on the electrode surface. According to these results, the produced MIP/GQDs/B-g-C_3_N_4_/GCE can be used successfully for the detection of BPA in real food samples.

Measurements were performed 30 times in succession in the presence of 0.5 nM BPA with only one prepared MIP/GQDs/B-g-C_3_N_4_/GCE. The RSD value of the calculated 0.44% verified the high repeatability.

A stability test of the BPA-imprinted sensor was conducted for 7 weeks (Figure S6). Little variation between the peak currents obtained over 7 weeks indicates the high stability of the developed sensor.

## 4. Conclusions

In conclusion, a novel molecularly imprinted electrochemical sensor based on GQDs/B-g-C_3_N_4_ nanocomposite was presented for bisphenol A detection. According to the experimental data, the doping of graphene quantum dots with boron-functionalized g-C_3_N_4_ improved importantly the electrochemical activity. The developed bisphenol A-imprinted sensor demonstrated a linearity (1.0 × 10^−11^–1.0 × 10^−9^ M) with a low LOD of 3.0 × 10^−12^ M, providing superior sensitivity and selectivity. This low LOD may be due to the following: (i) the doping treatment of GQDs and the boron element into g-C_3_N_4_ resulted in the specific surface area, indicating more interactions with BPA molecules; (ii) the synergistic effect between GQDs and the boron element increased easy electron transfer on the electrode surface. In conclusion, a new and efficient electroanalytical method was constructed for a sensitive bisphenol A sensor to obtain early disease diagnosis and safe food consumption.

## Figures and Tables

**Table 1:** Comparison of MIP/GQDs/B-g-C_3_N_4_/GCE with various methods.

Material	Linear Range (M)	LOD (M)	Ref.
UiO-66-NDC/GO	1.0 × 10^−5^–7.0 × 10^−5^	2.5 × 10^−8^	[38]
Au-MoO_3_/graphene	1.0 × 10^−8^–1.0 × 10^−4^	3.0 × 10^−9^	[39]
*Escherichia coli*/tyrosinase	1.0 × 10^−11^–1.0 × 10^−7^	1.0 × 10^−11^	[40]
Mo_2_Ti_2_AlC_3_ MAX phase/MWCNT	1.0 × 10^−8^–8.5 × 10^−6^	2.7 × 10^−9^	[41]
Carbon dot-V_2_O_5_	5.0 × 10^−9^–9.2 × 10^−3^	8.0 × 10^−10^	[42]
Iodine-doped graphene	4.0 × 10^−8^–4.5 × 10^−6^	2.0 × 10^−8^	[43]
MIP/GQDs/B-g-C_3_N_4_/GCE	1.0 × 10^−11^–1.0 × 10^−9^	3.0 × 10^−12^	This study

**Table 2:** Recovery results of BPA (*n* = 6).

Sample	Added BPA (nM)	Found BPA (nM)	* Recovery (%)
Orange Juice	-	0.203 ± 0.003	-
	0.100	0.304 ± 0.002	100.33 ± 0.02
	0.300	0.502 ± 0.001	99.80 ± 0.05
	0.500	0.701 ± 0.004	99.72 ± 0.04

* Recovery = Found BPA, nM/Real BPA, nM.

## Data Availability

The original data in this study are included in this study, and further inquiries can be directed to the corresponding author.

## Supplementary Materials

The following supporting information can be downloaded at: https://www.mdpi.com/article/10.3390/bios13070725/s1, Figure S1: (A) 100.0 mM Py polymerization including 25.0 mM analyte on GQDs/B-g-C3N4/GCE (Scan rate: 100 mV s^−1^), (B) Differential pulse voltammograms (DPVs) of the prepared electrodes in this study, (C) DPVs of different molecularly imprinting electrodes after rebinding of 0.5 nM BPA in 0.1 M PBS; Figure S2: The proposed electro-oxidation mechanism for BPA at GQDs/B-g-C3N4/GCE; Figure S3: AFM images (A) bare GCE and (B) BPA imprinted polymer film on GQDs/B-g-C3N4/GCE; Figure S4: Effect of (A) pH, (B) mole ratio, (C) desorption time, (D) scan cycle on signals of DPVs (in presence of 0.5 nM BPA) (n = 6); Figure S5: DPVs of (A) MIP/GQDs/B-g-C3N4/GCE and (B) NIP/GQDs/B-g-C3N4/GCE in 1.0 nM BPA, 100.0 nM HDP, 100.0 nM DOP, 100.0 nM 4-NIT, 100.0 nM ETH and 100.0 nM BPS; Figure S6: Figure S6. Stability test of MIP/GQDs/B-g-C3N4/GCE including 0.5 nM BPA (n = 6); Table S1: k and k′ values of BPA imprinted electrodes.
