# Supplementary material for: Bisphenol A Imprinted Electrochemical Sensor Based on Graphene Quantum Dots with Boron Functionalized g-C3N4 in Food Samples
Source: Biosensors (Basel). 2023 Jul 12;13(7):725. doi: 10.3390/bios13070725 (PMC10377542; doi:10.3390/bios13070725)
Supplement: Supplementary file 1 [file biosensors-13-00725-s001.zip › biosensors-2486137-supplementary.pdf]

Supplementary Data

For

Bisphenol A imprinted electrochemical sensor based on graphene quantum dots with boron functionalized g-C_3N_4 in food samples

Haci Ahmet Deveci [1], Müge Mavioğlu Kaya [2], İnan Kaya [3], Bahar Bankoğlu Yola [4], Necip Atar [5] and Mehmet Lütfi Yola [6,*]

Department of Nutrition and Dietetics, Faculty of Health Sciences, Gaziantep University, Gaziantep 27000, Turkey

[2] Department of Molecular Biology and Genetic, Faculty of Arts and Sciences, Kafkas University, Kars 36000, Turkey

[3] Department of Biology, Faculty of Arts and Sciences, Kafkas University, Kars 36000, Turkey

[4] Department of Engineering Basic Sciences, Faculty of Engineering and Natural Sciences, Gaziantep Islam Science and Technology University, Gaziantep 27000, Turkey

[5] Department of Chemical Engineering, Faculty of Engineering, Pamukkale University, Denizli 20000, Turkey

[6] Department of Nutrition and Dietetics, Faculty of Health Sciences, Hasan Kalyoncu University, Gaziantep 27000, Turkey

* Correspondence: mlutfi.yola@hku.edu.tr; Tel.: +90-3422118080; Fax: +90-3422118081

Apparatus

JEOL 2100 TEM (Tokyo, Japan), a Rikagu Miniflex X-ray diffractometer (Tokyo, Japan), Park FX40 AFM (Japan/USA) and a PHI 5000 Versa Probe type x-ray photoelectron spectrometer (Japan/USA) were used for TEM, XRD, AFM and XPS measurements. In addition, Gamry Reference 600 workstation (USA) was performed for the electrochemical investigations by using differential pulse voltammetry (DPV), electrochemical impedance spectroscopy (EIS), and cyclic voltammetry (CV).

Linearity

$$LOQ = 10.0\ S / m$$

$$LOD = 3.3\ S / m$$

S: Standard deviation of the intercept and m: Slope of the regression line

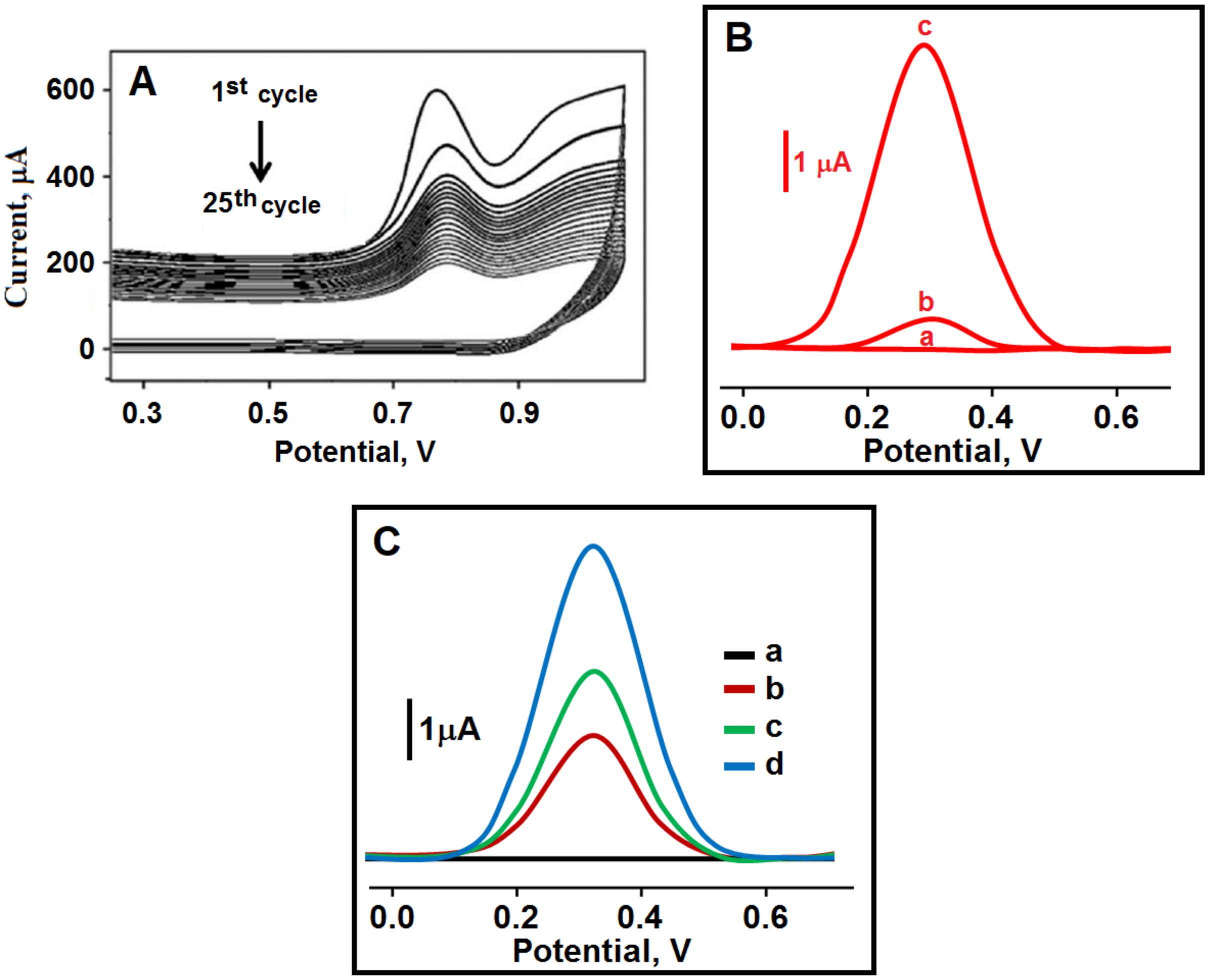

Figure S1. (A) 100.0 mM Py polymerization including 25.0 mM analyte on GQDs/B-g-C_3N_4/GCE (Scan rate: 100 mV s^{-1}), (B) Differential pulse voltammograms (DPVs) of the prepared electrodes in this study: (a) MIP/GQDs/B-g-C_3N_4/GCE in blank buffer solution (pH 7.0), (b) NIP/GQDs/B-g-C_3N_4/GCE after rebinding of 0.5 nM BPA in 0.1 M PBS (pH 7.0), (c) MIP/GQDs/B-g-C_3N_4/GCE after rebinding of 0.5 nM BPA in 0.1 M PBS (pH 7.0), (C) DPVs of different molecularly imprinting electrodes after rebinding of 0.5 nM BPA in 0.1 M PBS (a) MIP/bare GCE, (b) MIP/g-C_3N_4/GCE, (c) MIP/B-g-C_3N_4/GCE, (d) MIP/GQDs/B-g-C_3N_4/GCE.

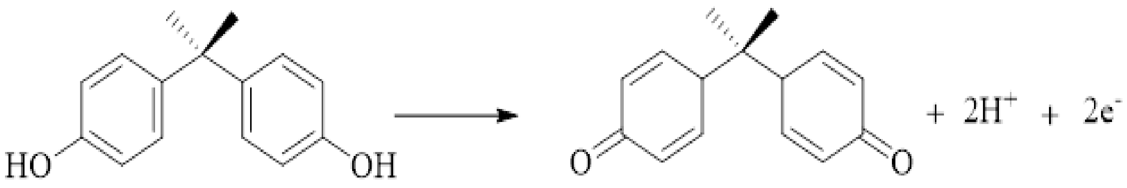

Figure S2. The proposed electro-oxidation mechanism for BPA at GQDs/B-g-C_3N_4/GCE.

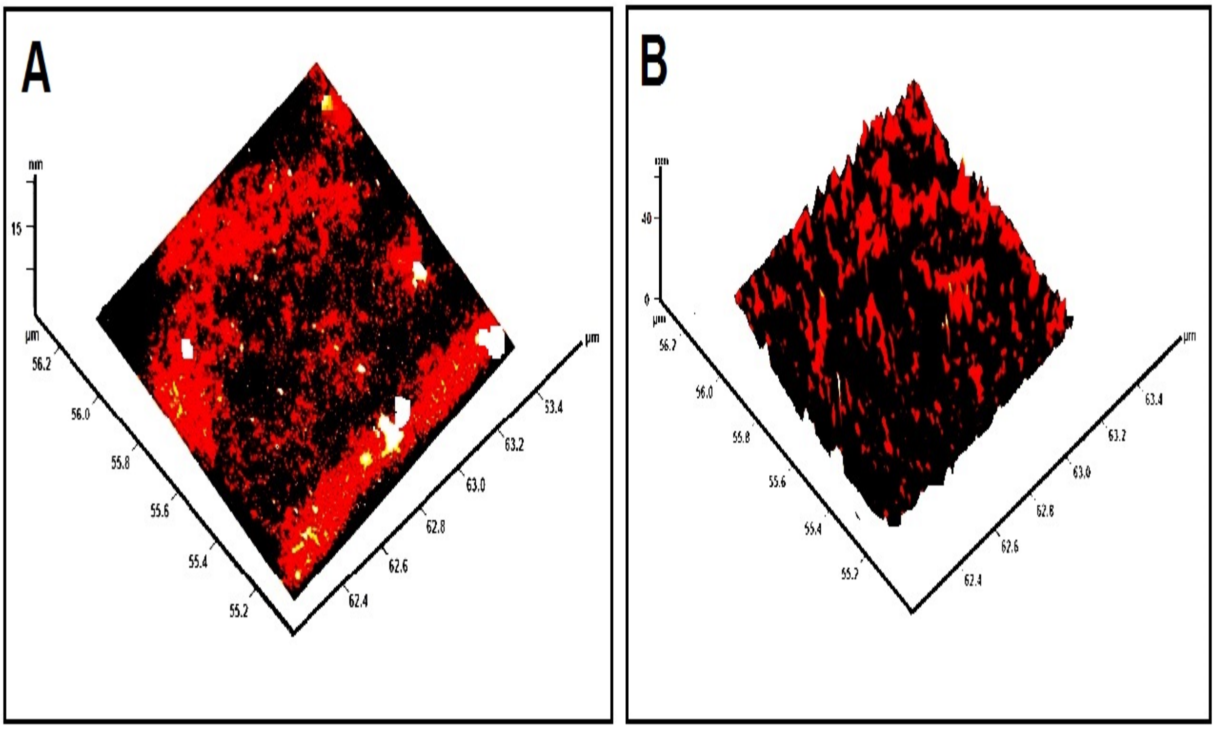

Figure S3. AFM images (A) bare GCE and (B) BPA imprinted polymer film on GQDs/B-g-C_3N_4/GCE.

3.4. Optimization studies

3.4.1. pH effect

The pH effect is an important factor that affects the sensor signals depending on the ambient conditions. When we looked at Fig. S4A, the sensor signal values increased up to pH 7.0 and then started to decrease at higher pHs. Thus, 7.0 was chosen as the ideal pH.

3.4.2. Mole ratio BPA to Py monomer effect

Secondly, mole ratio BPA to Py monomer effect was investigated (Fig. S4B). In the preparation of molecularly imprinted sensors, it is necessary to carefully adjust the monomer ratio. There is a high probability of non-specific interactions in large proportions of monomer concentrations. At low monomer concentrations, the number of binding sites of the analyte molecule is low and significantly affects the

sensitivity of the sensor. The highest sensor signal appears to be obtained when using 100.0 mM Py and 25.0 mM BPA.

3.4.3. Desorption time effect

It is very important to completely remove the analyte molecule from the electrode surface in the preparation of molecularly imprinted sensors. In the case of sensor preparation, the adsorption-desorption kinetics primarily affects the performance of the sensor. Consequently, a good optimization of the elution time is required. Several desorption times were tried for optimization and the most optimal signal value was obtained at 20 min (Fig. S4C).

3.4.4. Scan cycle effect

The thickness of the prepared electrode surface is very important in electrochemical sensor applications. Film thickness is an important factor that directly affects sensor performance. During the sonication process on the electrode surface with low film thickness, it is likely that the nanocomposite material will break off from the electrode surface. Therefore, molecularly imprinted electrodes with different film thickness were prepared and the highest signal signal was obtained in the electrode with 25th scan cycle (Fig. S4D).

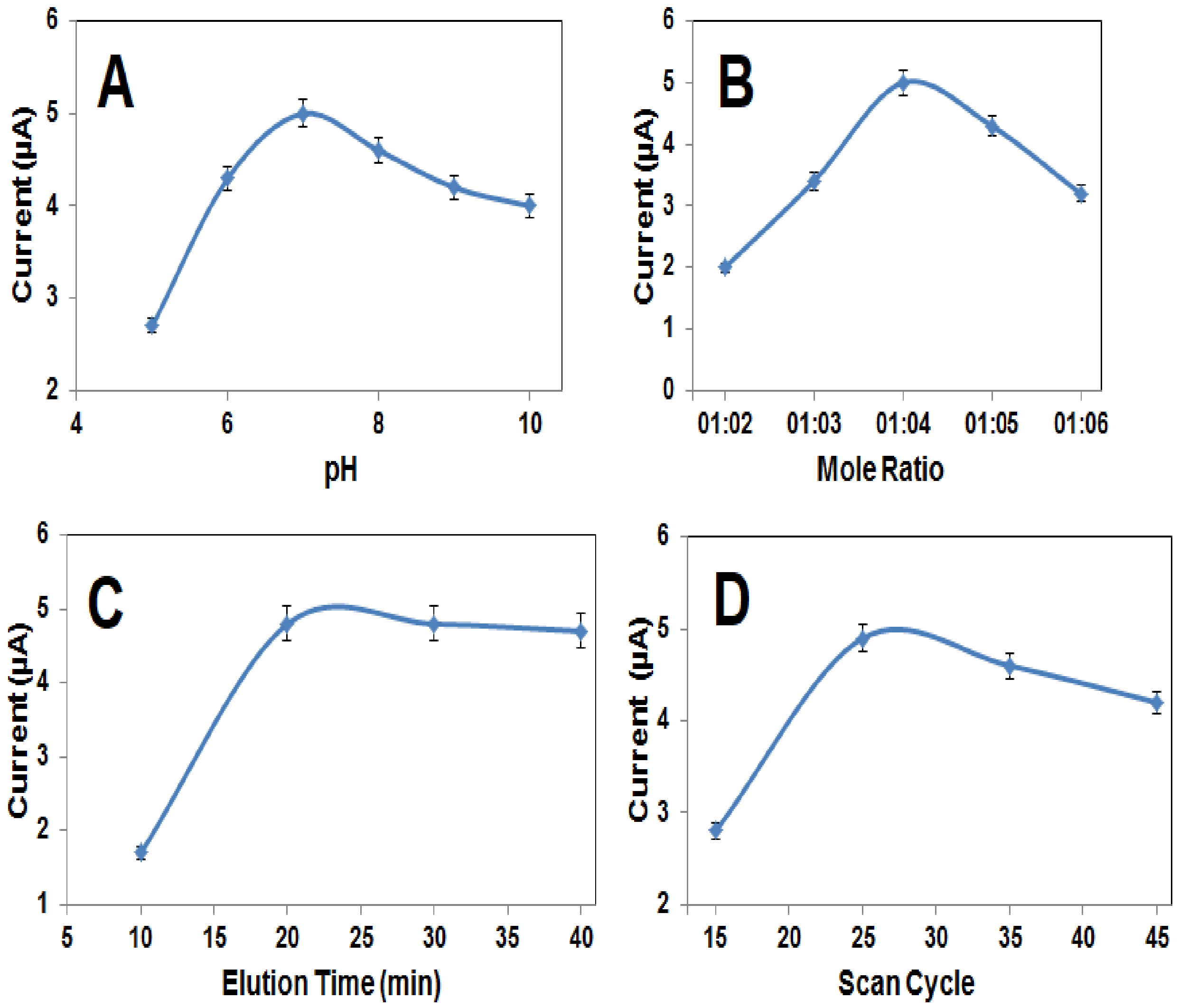

Figure S4. Effect of (A) pH, (B) mole ratio, (C) desorption time, (D) scan cycle on signals of DPVs (in presence of 0.5 nM BPA) (n = 6).

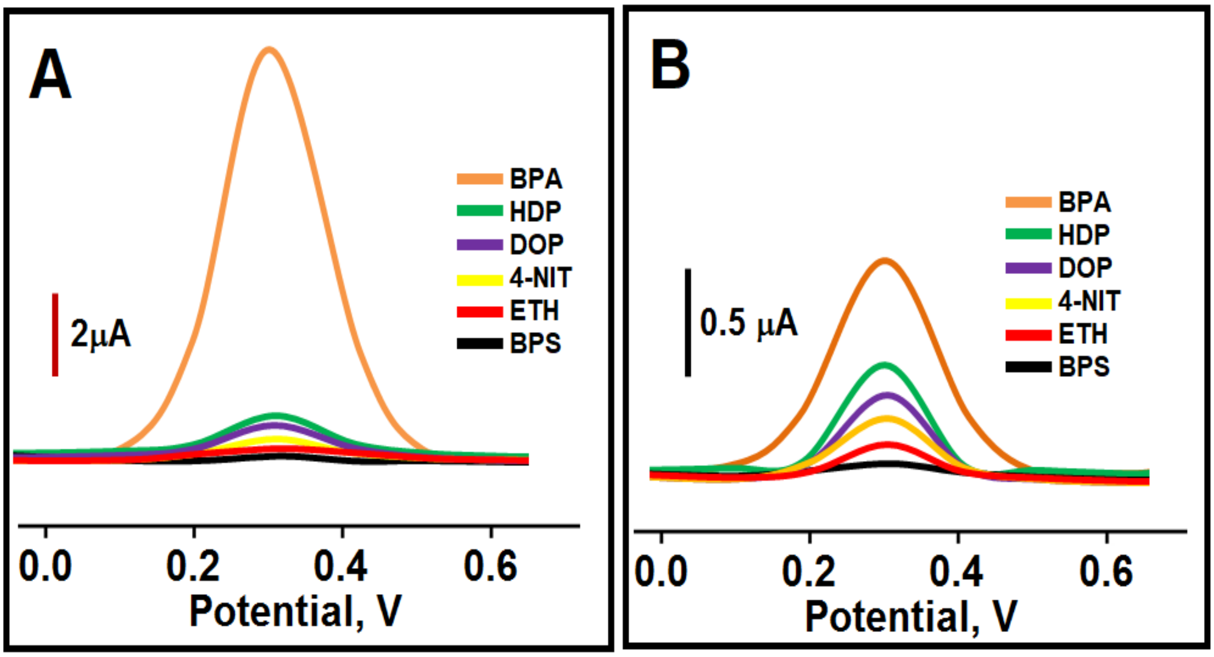

Figure S5. DPVs of (A) MIP/GQDs/B-g-C_3N_4/GCE and (B) NIP/GQDs/B-g-C_3N_4/GCE in 1.0 nM BPA, 100.0 nM HDP, 100.0 nM DOP, 100.0 nM 4-NIT, 100.0 nM ETH and 100.0 nM BPS.

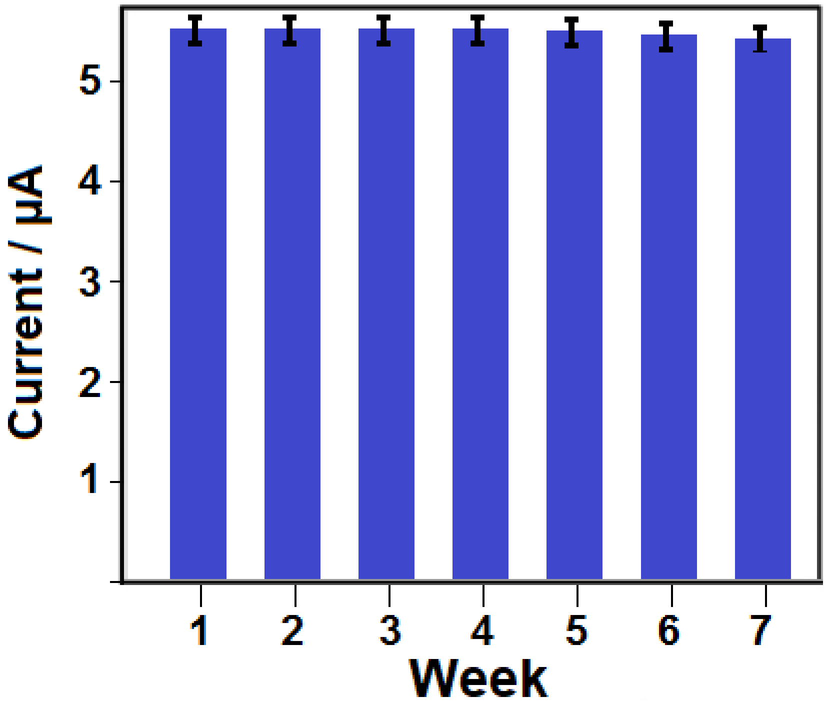

Figure S6. Stability test of MIP/GQDs/B-g-C_3N_4/GCE including 0.5 nM BPA (n = 6).

Table S1. k and k′ values of BPA imprinted electrodes (MIP/GQDs/B-g-C_3N_4/GCE and NIP/GQDs/B-g-C_3N_4/GCE)

	MIP		NIP		
	**ΔI (µA)**	**k**	**ΔI (µA)**	**k**	**k′**
BPA	10.00	-	1.00	-	-
HDP	1.00	10.00	0.50	2.00	5.00
DOP	0.75	13.33	0.40	2.50	5.33
4-NIT	0.50	20.00	0.30	3.33	6.01
ETH	0.25	40.00	0.15	6.67	6.00
BPS	0.10	100.00	0.05	20.00	5.00

Analyte concentrations: 1.0 nM BPA, 100.0 nM HDP, 100.0 nM DOP, 100.0 nM 4-NIT, 100.0 nM ETH and 100.0 nM BPS.
